# Beta-D-Glucan in Patients with Haematological Malignancies

**DOI:** 10.3390/jof7121046

**Published:** 2021-12-07

**Authors:** Malgorzata Mikulska, Elisa Balletto, Elio Castagnola, Alessandra Mularoni

**Affiliations:** 1 Division of Infectious Diseases, Department of Health Sciences (DISSAL), University of Genoa, 16132 Genoa, Italy; 2 Division of Infectious Diseases, IRCCS Ospedale Policlinico San Martino, 16132 Genoa, Italy; elisa.balletto@hsanmartino.it; 3 Infectious Diseases Unit, Department of Pediatrics, Istituto Giannina Gaslini, 16147 Genova, Italy; eliocastagnola@gaslini.org; 4 IRCCS-ISMETT (Istituto Mediterraneo per i Trapianti e Terapie ad Alta Specializzazione), 90127 Palermo, Italy; amularoni@ismett.edu

**Keywords:** glucan, invasive fungal infections, neutropenia, aspergillosis, pneumocystosis, cerebral infection

## Abstract

(1-3)-beta-D-glucan (BDG) is an almost panfungal marker (absent in zygomycetes and most cryptococci), which can be successfully used in screening and diagnostic testing in patients with haematological malignancies if its advantages and limitations are known. The aim of this review is to report the data, particularly from the last 5 years, on the use of BDG in haematological population. Published data report mainly on the performance of the Fungitell™ assay, although several others are currently available, and they vary in method and cut-off of positivity. The sensitivity of BDG for invasive fungal disease (IFD) in haematology patients seems lower than in other populations, possibly because of the type of IFD (lower sensitivity was found in case of aspergillosis compared to candidiasis and pneumocystosis) or the use of prophylaxis. The specificity of the test can be improved by using two consecutive positive assays and avoiding testing in the case of the concomitant presence of factors associated with false positive results. BDG should be used in combination with clinical assessment and other diagnostic tests, both radiological and mycological, to provide maximum information. Good performance of BDG in cerebrospinal fluid (CSF) has been reported. BDG is a useful diagnostic method in haematology patients, particularly for pneumocystosis or initial diagnosis of invasive fungal infections.

## 1. Introduction

Invasive fungal infections (IFDs) continue to be an important infectious complication in patients with haematological malignancy (HM) and those undergoing stem cell transplantation (HSCT). Early diagnosis and treatment can contribute to reducing the mortality, and over the past 20 years, significant efforts have been made to establish reliable diagnostic methods for IFD in the immunocompromised [1]. Together with early radiological evaluation, mycological assays, both culture-based and non-cultural have allowed prompt diagnosis of IFD in this setting.

Among non-culture-based assays, galactomannan (GM) detection in serum or in bronchoalveolar lavage (BAL) is the mainstay of the diagnosis of invasive aspergillosis. Although its performance is not perfect, it is the most important fungal marker in patients with HM, since aspergillosis is the most frequent IFD, while *Candida* infections are rather rare due to the common use of azole prophylaxis.

Another fungal marker is (1-3)-beta-D-glucan (BDG), which can be detected in serum for the diagnosis of not only aspergillosis but also other IFDs. The usefulness of BDG in the haematology setting has been extensively studied, with certain differences emerging for this population. The epidemiology of IFD is characterized by the predominance of invasive aspergillosis (IA) and a limited number of cases of invasive candidiasis (IC) as compared to other populations that are at risk for IC, such as intensive care unit (ICU) critically ill patients [2]. *Pneumocystis jirovecii* pneumonia (PJP) is another important IFD in the haematology population, for which BDG has been shown useful.

However, the availability of BDG is much more limited than that of GM, as shown in a recent survey on the treatment of IA in haematology patents in Europe [3]. While GM was available in serum and bronchoalveolar lavage in most of the 112 included centres (95% and 87%, respectively), BDG was available only in 21%. Thus, the practical knowledge of this test, including its strengths and limitations in the haematology setting, is fundamental for deciding whether to add it to the diagnostic armamentarium in single haematology centres.

BDG can be a valuable contribution to the diagnosis and management of IFDs in the haematology setting, provided clinicians have thorough knowledge of the advantages and limitations of the use of the BDG test in this specific population, which are different from other patient populations such as critically ill or surgical patients.

The aim of this review was to provide an update on recent data on the utility of BDG in the haematology setting.

Therefore, an updated MEDLINE/PubMed search (search terms: glucan AND [hematolog* OR haematolog* OR neutropeni*]) was performed on 1 October 2021, limited to the years 2016–2021. Previous relevant data were also considered.

## 2. Description of BDG Assays

The BDG test is based on the detection of the polysaccharide (1,3)-ß-D-glucan, which is a component of the cell wall of many pathogenic fungi, except for Mucorales and most strains of *Cryptococcus* [4,5]. Since BDG can be positive in numerous different IFDs, other diagnostic methods, both radiological and mycological, are necessary to attribute a positive BDG result to a specific IFD.

While the first BDG assay was introduced in the 1980s in Japan, other assays were developed and are currently approved by U.S. Food and Drug Administration or carry the Conformite Europeenne (CE) marking, with the Fungitell assay (Associated Cape-Cod, Inc., East Falmouth, MA, USA) being the first one approved by the US FDA in 2004 and then marked CE, and most comparisons performed between Fungitell and Wako assays (Wako Pure Chemical Industries, Tokyo, Japan) [6,7]. These assays have different methods for BDG detection and cut-off values for positivity, which include, in some assays, the indeterminate zone. The detailed characteristics of the assays are outlined in Table 1 [7,8,9,10,11].

## 3. Recent Data on Optimized Thresholds

Recently, a prospective comparison of Fungitell and Wako resulted in a proposal of optimized thresholds (higher and lower than the manufacturer’s recommendations, respectively) [12]. The study was performed in 171 patients, mainly with HM (62%), who had experienced 175 episodes of suspected IFD, with the final diagnosis of 23 infections due to BDG-producing fungi (including 12 cases of PJP). For optimized thresholds of 120 pg/mL for Fungitell and 4 pg/mL for Wako, the sensitivity and specificity were 82% and 95% for Fungitell and 82% and 95% for Wako, compared to a Wako sensitivity of 50% with the manufacturer’s positivity threshold of 11 pg/mL and a specificity that remained similar [12].

In a recent retrospective analysis of serum samples from patients with and without PJP based on clinical and radiological characteristics and polymerase chain reaction (PCR) in BAL, the performance of Fungitell and Wako assays was compared [13]. At the manufacturer’s recommended cut-offs (80 pg/mL and 11 pg/mL, respectively), the Wako assay was found significantly more specific (0.98 vs. 0.87) and the Fungitell assay more sensitive (0.78 vs. 0.85), with similar overall performance. At a cut-off of 3.616 pg/mL, the Wako assay had similar sensitivity to the Fungitell assay (0.88 at a cut-off of ≥60 pg/mL), but its specificity was significantly higher (0.89 vs. 0.82) [13].

In conclusion, a higher cut-off for the Fungitell and lower for the Wako assay might result in improved overall performance in patients with HM. However, reducing the sensitivity of Fungitell, which is the advantage of this assay, might be counterproductive in the haematology setting, especially in the diagnosis of IPA or pre-emptive antifungal therapy for febrile neutropenia, when sensitivity is crucial [14]. In these cases, lower specificity of standard cut-offs might be preferable, and repeated sampling might rule out false positive results.

## 4. False Positive and False Negative Results of BDG Assay

All the reported causes of false positive and false negative results of BDG are reported in Table 2 [2,15,16,17,18,19,20,21,22,23,24,25,26,27]. Interestingly, recently reported sporadic causes of false positive BDG results include intake of seaweed in a HSCT recipient with grade II skin and gastrointestinal graft versus host disease (GvHD) (with a maximum BDG value of 229 pg/mL) [28] and continuous administration of penicillin G (with a maximum BDG value of 81 pg/mL) [29].

## 5. BDG in Screening in Patients with Haematological Malignancies

Numerous studies have reported experience with BDG screening in haematological populations, some of them reporting better performance if used with other markers, such as GM [30]; however, the results varied based on the prevalence and type of IFD in the screened population and the study design (case-control vs. cohort studies): in the case control studies, the group of “grey area” patients in whom IFD could not be confirmed or excluded were not included.

Indeed, in a large, recent study of BDG in screening and diagnosis in haematologic patients receiving anti-mould prophylaxis, i.e., with low prevalence of IFD, among the 203 episodes included, 62 were classified as non-evaluable [18]. In that study, eight episodes of proven/probable IFD were diagnosed (5.7%), and the sensitivity, specificity, positive predictive value, and negative predictive value were 50.0%, 84.2%, 16.1%, and 96.5%, respectively. Despite high NPV (negative predictive value), with a sensitivity of 50%, the use of BDG to exclude IFD does not seem appropriate outside the setting with a very low pre-test probability.

Therefore, the meta-analysis performed for the European Conference on Infections in Leukemia (ECIL) and published in 2012 is very helpful, as it attempted to limit this heterogeneity of the studies by restricting the analysis to prospective cohort studies (in 5/6 of which BDG was used for screening, and in 1/6 for targeted diagnosis) in 1771 haematology patients [31]. For the diagnosis of proven or probable IFD, the positive test sensitivity and specificity were 70% (95% CI 47–86) and 91% (95% CI 83–96), respectively, while for two consecutive positive tests, they were 50% (95% CI, 34–65) and 99% (95% CI, 99–99.5), respectively [31].

In addition, in our personal experience with BDG screening in 167 neutropenic patients with HM, of whom 20 (12%) developed invasive aspergillosis, the sensitivity was suboptimal [32]. In the per-patient analysis, sensitivity and specificity for a single positive result were, respectively, 60% and 78%, and 40% and 93% for two consecutive positive results. Such high specificity was similar to that of serum GM (90%), and in per-sample analysis, the specificity of BDG was 89–100%, highlighting that in the haematology setting, persisting false positive results are not as frequent as feared. Of note, BDG became positive before GM in one-third of patients, pointing to different kinetics of fungal markers and the possibility of earlier diagnosis if more than one marker is used [32].

Similarly, in a prospective study that evaluated a combination of fungal biomarkers in 135 haematology patients, with 13 cases of IFD (10%), the sensitivity of BDG (the Glucatell assay) at the time of diagnosis was rather low, at 69% (95% CI 42–87), but increased to 92% when evaluated within 2 weeks of the time of diagnosis. The specificity for the cut-off of 80 pg/mL was very low (41%, 95% CI 32–50) but dramatically increased if two consecutive positive results were considered (80%, 95% CI 72–87), without any reduction in sensitivity. Interestingly, of the various antifungal markers included, BDG was the one with best performance [33].

In conclusion, in haematology patients, serum BDG can be used in screening, keeping in mind that a negative result does not exclude the presence of IFD, and repeated testing might be required, while two consecutive positive results are associated with very high probability of true positivity and should warrant complete diagnostic workup and therapeutic decisions.

**Figure 1:**
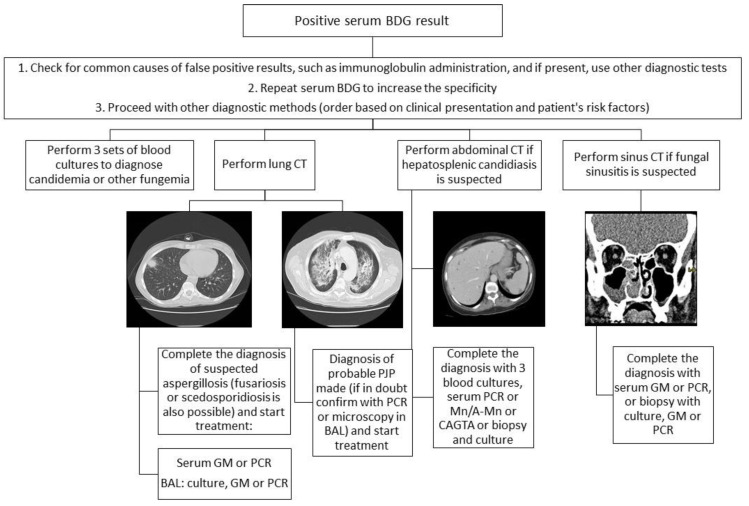
Practical approach to the use of a positive BDG result in patients with HM (BAL, bronchoalveolar lavage; CAGTA, *Candida albicans* germ-tube antibody IgG assay; CT, computed tomography; Mn/A-Mn, mannan antigen and anti-mannan antibodies; PCR, polymerase chain reaction).

A practical approach to the use of a positive BDG result in patients with HM is shown in Figure 1.

## 6. BDG in Diagnosis of Invasive Candidiasis and Invasive Aspergillosis

There are no data in haematology patients specifically on the performance of BDG in candidiasis, possibly because this IFD is not very frequent in patients routinely undergoing screening with fungal markers due to *Candida*-active prophylaxis. Indeed, the incidence rates of candidemia and chronic disseminated candidiasis in 3027 patients with HM ranged between 0.74–0.77 and 0.30–0.44 according to the group of patients [34]. However, comparison of the performance of BDG in different IFDs has been reported in recent studies.

In the study by Alanio and colleagues, which focused on the comparison of two different BDG assays, the sensitivity of both assays for the diagnosis of IA was significantly lower than for candidiasis or PJP: 57% (95% CI 18–90) for IA vs. 72% (95% 39–94) for IC vs. 79% (95% 49–95) for PJP for the Fungitell assay [12].

Similar results were observed in a study that included 143 patients with proven or probable IFDs (49 cases of IC, 45 IA, and 49 rare IFDs) and analysed BDG with the Fungitell assay. At the time of diagnosis, the sensitivity of BDG was 64% in patients with candidemia, 52% in those with probable/proven IA, and 61% in those with rare IFDs [35]. There was no correlation between negative BDG results and patients’ characteristics, localization of infection, or prior antifungal use, but the sensitivity was higher in the case of *C. albicans* infections compared to other *Candida* species.

In a recent prospective Australian experience of BDG screening of 57 episodes in 52 haematology patients at high risk of IFD, eight episodes of proven/probable IFD and 18 episodes of possible IFD were diagnosed [36]. Most of the patients received antifungals, either in prophylaxis or treatment. Overall, BDG resulted positive in 32% of samples, but the sensitivity of BDG for proven/probable IFD was only 5/8 for a single positive sample and 3/8 for two consecutive positive samples, while BDG was positive in the absence of IFD (false positive) in 8/11 episodes. The reasons for such a poor performance remain unknown, with cyclosporine and methotrexate therapy found associated with false positive results, possibly due to circulating endogenous glucans from increased gut permeability [36]. Low sensitivity could be influenced by the high percentage of patients (63.2%) receiving anti-mould-active antifungal agents, including all patients who developed proven/probable IFDs. However, this was not assessed, as a comparator group was lacking.

## 7. BDG in Diagnosis of Pneumocystosis

The incidence of PJP has been increasing in immunocompromised patients such as haematopoietic stem cell or solid organ transplant recipients, patients with autoimmune or rheumatic immune diseases, and patients undergoing systemic chemotherapy [37,38]. Mortality rates are significantly higher in immunocompromised non-human immunodeficiency virus (HIV) patients [39], possibly due to delayed diagnosis, more severe disease at presentation, greater medical complexity of the patient, and competing risk of death from underlying illnesses [40].

Among the factors contributing to increased PCP-related mortality in non-HIV-infected individuals is timely diagnosis; this is crucial for the improvement of PCP-related prognosis. Based on the Revised EORTC/MSGERC Invasive Fungal Disease Definitions for *Pneumocystis jirovecii* Disease in Individuals Without Human Immunodeficiency Virus, the diagnosis of proven PCP is based on clinical and radiologic criteria plus demonstration of *P. jirovecii* by microscopy using conventional or immunofluorescence staining in tissue or respiratory tract specimens [1]. Probable PJP is defined by the presence of appropriate host factors and clinical-radiologic criteria, plus amplification of *P. jirovecii* DNA by quantitative real-time PCR in respiratory specimens and/or detection of β-d-glucan in serum, provided that another invasive fungal disease and a false-positive result can be ruled out. The inclusion of the serum BDG test is based on high sensitivity and excellent negative-predictive value; uniformly accepted thresholds, however, have not been defined [41].

BDG is a non-invasive test to support the diagnosis of PJP, especially in situations where critical illness precludes invasive diagnostic procedures [42]. Given its high sensitivity, a negative BDG result has been proposed as an important test that could contribute to rule out a diagnosis of PJP; in ECIL guidelines for patients with HM, negative serum BDG was considered (with the grading of A II) sufficient to rule out PJP [43]. This recommendation was based on a very high negative predictive value in two meta-analyses, which was >94% even in the case of a prevalence of PJP of 50% [43]. However, studies that have demonstrated that a high level of BDG is a discriminative marker of PCP in immunocompromised patients were mainly focused on HIV-infected patients [44,45,46] or on mixed populations of HIV and non-HIV infected patients [46,47,48,49,50,51,52]. There are fewer studies only involving non-HIV patients.

In fact, some recent studies have highlighted lower than previously reported sensitivity of BDG in non-HIV patients with PJP. The study by Damiani and colleagues evaluated the performance of BDG assay in 39 patients with systemic autoimmune or inflammatory disorders, solid organ transplant, hematologic malignancy, or cancer with proven PJP, as well as 39 *Pneumocystis*-colonized patients (defined as positive PCR assay, negative microscopy, and clinical improvement in the absence of pneumocystis-active treatment) matched by an underlying condition. In this study, the BDG test had a sensitivity of 87%, specificity of 97%, positive predictive value of 97%, and negative predictive value of 88%. The median BDG level was lower in the group of PJP patients with hematologic malignancy (211 pg/mL) compared to the levels observed in solid organ transplant patients (3473 pg/mL) or in patients with autoimmune or inflammatory disorders (3480 pg/mL) with proven PJP. Indeed, the authors concluded that in patients with HM, with a BDG sensitivity of 64% and negative predictive value of 73%, a negative BDG result alone is not sufficient to rule out PJP [53]. Similarly, in 18 patients with HM and proven/probable PJP, median BDG levels were high in those with proven (1108 pg/mL) and probable (612 pg/mL) infection, making BDG potentially helpful, but the sensitivity (83–89%) was considered insufficient to exclude PJP [47]. Additionally, in a retrospective study of 31 HIV-positive and 44 non-HIV patients with PJP, BDG positivity was 72.7% in non-HIV vs. 93.5% in HIV groups (*p* = 0.034) [54].

Finally, a systematic review and meta-analysis of the utility of serum BDG testing, studying 997 patients with PCP and 3062 controls, reported significantly lower sensitivity in non-HIV patients compared to the HIV population (94% vs. 86%), with a similar specificity of 79% (95% CI, 72–84%) [52]. In fact, the authors concluded that a negative BDG test is only associated with a low post-test probability of PCP (≤5%) when the pre-test probability is low (≤20%) in patients without HIV [52]. These differences might be due to the fact that the burden of *P. jirovecii* organisms in the lungs of HIV patients with PJP is higher than that in non-HIV PCP patients [55].

The results of recent studies on the performance of BDG in PJP, with particular attention to the cut-off used and the comparator group, are presented in Table 3 [42,47,53,56].

Another interesting issue is the potential of serum BDG to distinguish between infection and colonisation in Pneumocystis-PCR positive patients. A retrospective study performed in a mixed population of 166 HIV and non-HIV patients (patients with HM, collagen vascular disease, solid tumour, and organ transplantation) found that BDG levels (measured with the Wako assay) in the definite PJP group were significantly higher than those in the groups of negative, colonization, and probable PJP (all *p* < 0.001) [57]. Serum BDG levels in patients with definite/probable PJP were also significantly higher than those in patients with colonization who had positive PCR results but improved without anti-pneumocystis treatment. The cut-off level for discrimination was estimated at 33.5 pg/mL, and the authors suggested that a positive BDG result might be a good indicator for beginning anti-PJP treatment [57]. Additionally, in solid cancer and hematologic malignancy patients with unexplained lung infiltrates, higher BDG values (>200 pg/mL with Fungitell assay) were consistently associated with clinical PJP among BAL PCR-positive patients, while patients with negative BDG and PCR were unlikely to have PJP (for details on different cut-offs, see Table 3) [42].

As far the correlation between BAL quantitative Pneumocystis-DNA PCR and serum BDG in various patient populations is concerned, a good correlation can be obtained in HIV patients and solid organ transplant recipients, but no correlation was observed in patients with hematologic malignancies, solid cancer, and systemic diseases. This observation added to recent data suggesting that BDG is not the perfect marker of PJP in non-HIV patients, with potential false positives due to other IFDs or bacterial infections and false negatives due to low fungal load and low BDG release [58].

It is still undefined whether serum BDG can reflect the severity or prognosis of PJP infection and predict treatment response or outcome [59].

In conclusion, parallel to the increasing incidence of non-HIV immunocompromised patients with PJP, the data from this population on BDG have increased, highlighting that there may be important differences in BDG test performance between different subsets of patients. In general, BDG detection for PJP diagnosis has adequate sensitivity and can provide helpful diagnostic support, especially when invasive testing for PCP is not feasible. A positive BDG result should trigger tests to also exclude other IFDs in these vulnerable immunocompromised patients, while a negative result in a patient with HM with compatible clinical presentation warrants the confirmation of the absence of PJP with negative BAL PCR or microscopy.

Additionally, in the evaluation of the performance of BDG, the “comparator test” differed across the various studies, so the new definition provided by EORTC/MSGERC might be helpful to uniformly define proven and probable populations [1]. Last but not least, different cut-offs of various commercial assays used to define positivity might further affect studies’ comparability.

## 8. BDG in Other Invasive Fungal Diseases

BDG has been reported to be positive in numerous other fungal infections, including the more common fusariosis or scedosporidiosis and the rare *Exserohilum rostratum*, a fungus associated with the widespread outbreak of iatrogenic CNS infections [60].

Two recent papers have highlighted its use in fusariosis. In a retrospective study of 13 patients, BDG was positive in 12 patients (92%), in a median of 10 days (range 1–32) before the time of diagnosis [61]. Unfortunately, there was also a very high rate of positivity in the 13 selected cohorts, yielding a PPV of 7%. In addition, in a cohort of eight cases of fusariosis in Spain, beta-D-glucan was positive in 100% of cases, while GM only in 37.5% [62].

Interestingly, in an outbreak of six cases of *Saprochaete clavata* infections (a fungus formerly called *Geotrichum clavatum* and known for its resistance to echinocandins), BDG was positive in three patients, with the serum positivity levels ranging from 159 to >523 pg/mL [63].

## 9. BDG in Samples Other Than Serum

Currently, BDG is only approved for the use in serum; however, testing in other samples, sterile and not sterile, has been studied and successfully used in clinical practice.

Among them, the performance in cerebrospinal fluid (CSF) is particularly interesting. Although not limited to haematology patients, a systematic review of BDG in CSF included 14 studies and various fungi such as *Candida*, *Aspergillus*, *Exserohilum*, and *Histoplasma* [64]. Fox example, for *Histoplasma* meningitis, BDG was found to have 53% sensitivity and 87% specificity, while for *Exserohilum rostratum* it was 100% and 98%, respectively, for the cut-off of 138 pg/mL. Although most cryptococci are thought to not contain BDG, in an HIV-positive cohort with cryptococcal meningitis, CSF BDG sensitivity was 89% and specificity 85% [65].

On the contrary, the detection of BDG in BAL has been repeatedly found to have very poor specificity and is therefore of little use [66,67]. Its potential to be used to rule out aspergillosis, which has been proposed for the ICU setting, should be confirmed in other studies [66].

## 10. BDG in Children with Haematological Malignancies Undergoing Antineoplastic Chemotherapy or Stem Cell Transplantation

Recent review papers [68,69,70] showed a poor performance of the test in paediatrics, unless using a cut-off much higher than the 80 pg/mL, indicated as a threshold for a positive test [68]; it is discouraged for prospective monitoring or diagnostic use in paediatric patients at high risk of invasive fungal disease in the most recent guideline on management of IFD in children receiving chemotherapy or SCT (grade D recommendation, level of evidence II) [71]. Recently, BDG has been proposed in combination with GM and *Aspergillus* PCR for the diagnosis of invasive aspergillosis in paediatric patients [72]. The performance of the test was improved using a higher cut-off (>300 pg/mL) and using the test only after the acute post-transplant phase, where a high proportion of false positive results were observed [72].

## 11. BDG for Monitoring of Response in Invasive Fungal Diseases

There are limited data on the kinetics of BDG decline in the case of IFDs. Although a negative slope in BDG levels correlated with successful outcome in patients with invasive candidiasis, the rate of decline might vary significantly, and values above the positivity threshold may persist after clinical cure and be of little prognostic value within a clinically meaningful timeframe [73,74].

However, some positive experiences have been recently published. In the already mentioned study of 143 patients with different IFDs, in 70% of the patients with a follow-up, BDG became negative at more than 1 month for candidemia and more than 3 months for IA, and slower BG decrease in patients with candidemia was associated with deep-seated localizations [35]. The potential usefulness in deep-seated infections was also illustrated by a recent case of *Aspergillus* ventriculitis, in which BDG was successfully used to monitor the response and to guide the length of antifungal therapy, which was discontinued after two consecutive negative CSF BDG measurements a 4-week period [75]. In that case, CSF was positive for BDG at almost 2 years, with levels declining to <200 pg/mL after the first 10 months of treatment. Similarly, in the case of *Exserohilum rostratum* meningitis, the persistently high levels of BDG in CSF fluid were associated with poor clinical outcome [60].

In a population of haematology patients with candidemia (*n* = 28) and chronic disseminated candidiasis (*n* = 12), BDG sensitivity was, respectively, 43.5% and 73%, but its kinetics correlated with the clinical outcome, with a sharp decline in BDG levels in those with resolution of candidemia and a decrease in BDG levels within 2 to 6 months in patients with chronic candidiasis who survived. Of note, BDG negativisation preceded resolution of lesions on CT [34]. In contrast, in another recent case of proven hepatic candidiasis, the positivity of BDG lasted less than 20 days, with an important decrease 10 days after the onset of antifungal therapy [76]. In the already mentioned study in patients with fusariosis, BDG increased in patients who died by day 30, but remained stable during this timeframe in those who survived [61].

On the other hand, there were also reported cases of prolonged BDG positivity months after the clearance of candidemia in HSCT recipients without any other IFD and in whom the central venous catheter had been removed [77,78].

## 12. Serum BDG in the Diagnostic Criteria of Invasive Fungal Diseases

The contribution of the BDG test in the diagnosis of IFD in haematology patients has also been highlighted in the guidelines of the Third European Conference on Infections in Leukemia (ECIL-3), in which the use of BDG was given a B-II grading of recommendation for the diagnosis of IFD (moderate evidence for use) [31].

ESCMID guidelines recommend, with grade C II, the use of serum BDG to diagnose IFD and to diagnose and screen for IA in adults with HM and after stem cell transplant [79].

BDG was included in the first revision in 2008 of EORTC/MSG criteria for the diagnosis of IFD in the immunocompromised as a mycological criterion for the diagnosis of any probable invasive fungal disease other than cryptococcosis and mucormycosis [80]. In the second revision of the EORTC/MSG diagnostic criteria in 2019, serum BDG (two consecutive samples above 80 pg/mL for Fungitell assay) is considered as a mycological criterion only of probable invasive candidiasis or probable pneumocystosis, but not among the criteria for the diagnosis of invasive aspergillosis or other invasive mould infections [1]. This is the first time that these criteria include also pneumocystis, and in case of this IFD, both positive serum BDG and molecular detection of Pneumocystis-DNA in BAL are considered as criteria of probable infection, while microscopic detection is required for the diagnosis of proven PJP, and the basis for such classification was reviewed separately [41].

## 13. Summary of Strengths and Limitations of BDG Assay

The correct use of BDG in clinical practice requires the knowledge of its strengths and limitations, which are summarized in Table 4.

## 14. Conclusions

BDG is a versatile test, which can be successfully used in screening and diagnostic testing in patients with HM in combination with clinical assessment and other diagnostic tests, both radiological and mycological. There may be variability in PPV and NPV, based on patient population and type of IFD, and lower sensitivity if case of IA, compared to IC and PJP might be expected. In the haematology setting, NPV is insufficient to exclude diagnosis of IFD. The specificity of the test can be improved by using two consecutive positive assays and avoiding testing in the case of the concomitant presence of factors associated with false positive results, such as immunoglobulin administration. In addition, serum good performance of BDG in CSF was reported. The real-life performance of various new marketed assays will need to be determined.

## Figures and Tables

**Table 1:** Comparison of characteristics of different beta-D-glucan test assays.

Test Assay	Availability	Producer	Method	Cut-Off Value	Overall Sensitivity	Overall Specificity	Comments	Reference
Fungitell	Europe, US	Associates of Cape Cod, Falmouth, MA, USA	Colorimetric	Intermediate 60–79 pg/mL Positive > 80 pg/mL	27–100%	0–100%	BDG detection by Fungitell is part of EORTC/MSG criteria (positive serum BDG in combination with host factor and clinical criterion) for probable invasive candidiasis or PJP.	[7,8,9]
Fungitell STAT	Europe, US	Associates of Cape Cod, Falmouth, MA, USA		Indeterminate 0.75–1.1 Positive ≥ 1.2	ND	ND	New rapid test that can be run on one or more patient specimens (single sample testing), but initial clinical validation reported 98–99% concordance with indeterminate results excluded and 74–91% if included.	[11]
Glucatell	Europe, US	Associates of Cape Cod, Falmouth, MA, USA	Colorimetric	80 pg/mL	50–92%	41–94%	The Glucatell test differs from the Fungitell test in that the Glucatell reagent is processed to eliminate Factor C. This makes the Glucatell test more specific for BDG linkages. The Glucatell reagent does not react to other polysaccharides, including beta-glucans with other glycosidic linkages.	[7,9]
Wako Wako-EU	Asia, Europe	Wako Pure Chemical Industries, Osaka, Japan	Turbidimetric method	11 pg/mL	50–86%	89–100%		[7,8,9,10]
Fungitec G test ES Fungitec G test MKII	Europe, US	Seikagaku Kogyo Corporation, Tokyo, Japan Subsidiary Associates of Cape Cod, Falmouth, MA, USA	Colorimetric method	20 pg/mL	67–88%	60–85%		[7,9]
Dynamiker Fungus	Some European countries and North Africa	Dynamiker Biotechnology Ltd., Tianjin, China	Turbidimetric method	95 pg/mL	64–81%	78–80%		[7,9]

ND, no data.

**Table 2:** Potential causes of false positive and false negative results of serum BDG; underlined cases are considered as clinically important and potentially frequent [2,15,16,17,18,19,20,21,22,23,24,25,26,27].

FALSE POSITIVES	Mechanism	Comments
**Iatrogenic contamination**		
Haemodialysis	Use of regenerated cellulose dialysis membrane	Modern dialysis membranes (non-BDG-leaching synthetic membranes) no longer release BDG, and BDG was highly specific for the diagnosis of IFD in the serum of patients receiving haemodialysis in a recent study [27].
Blood and blood derivates such as immunoglobulins and albumin	Cellulosic depth filters are generally mixtures of cellulose and diatomaceous earth and are used to provide initial clarification of blood plasma. Process solutions may also contain BDG and introduce contamination.	The risk of false positivity after receiving blood or blood components seems dependent on the product’s concentrations of BDG and is not constant (for example, never observed in our hospital), while immunoglobulin preparations almost invariably contain BDG [17]. These high titres usually decline rather quickly, and such responses support suspicion of iatrogenic contamination. The depth filters flush strategy was developed to control beta-glucan leaching into the product pool [23].
Cellulose containing gauzes/surgical sponges		The release of BDG from surgery gauzes is temporary and depends on the type of gauze used [25].
Non-glucan-free laboratory equipment		Currently unlikely, since glucan-free laboratory equipment is available.
Beta-lactam antibiotics (e.g., ampicillin-sulbactam, amoxicillin-clavulanate)	BDG may be present in the original source material itself, such as products made by fungal fermentation, in excipients added to the formulation, from media used in microbial or cell culture, or from process equipment, materials, and solutions.	Possible; however, the high level of dilution generated upon injection of relatively low volumes of antibiotic make this unlikely. Further, the high negative predictive value for IFD observed for patients receiving a vast array of antibiotics suggests that this is not a significant problem [20].
**Intestinal translocation**		
Bacteriemia	Translocation as a consequence of ischemic damage to the intestinal barrier due to septic shock	Some experiences suggest that bacteraemia is a very rare source of false positivity [21].
Severe mucositis	Possible translocation of fungal antigens through the intestinal mucosa damaged by chemotherapy	Whether or not this might truly affect specificity of BDG in adult hematologic patients remains controversial, but should be considered in patients with intestinal GvHD or severe mucositis [2].
Major abdominal surgery	Translocation as a consequence of loss of integrity of the intestinal wall	Rare in haematology.
Gut ischemia	Translocation as a consequence of ischemic damage	
Burns	Large surface area burns	Validation of alternative cut-offs in specific clinical contexts known to contribute to elevated BDG titre may provide the solution to specificity issues [24]. Unknown if applicable also to severe skin acute GvHD.
Chronic kidney disease	Uremia’s metabolic toxicity	
*Enterococcus* spp. bacteremia	Protease-producing intestinal enterococci	
**Hepatic function**		
End-stage liver disease	Reduced clearance	
**Bacterial infections**		
*Nocardia* spp. infection		Although rare, needs to be considered in differential diagnosis in case of compatible clinical presentation (pulmonary, cerebral) [15].
*Streptococcus pneumoniae* Type 37	Producing a BDG with a (1→3)-β-backbone [15]	
*Pseudomonas* spp.	Producing (1→2)-β-linked glucan sequences [15]	
**Interference**		
Pegylated asparaginase	Drug-related alterations in heme metabolism, which in turn interfere with measurement of BDG in serum [2]	
Haemolysis	Interference with test procedure.	Possible interference, particularly for colorimetric assays [22].
**FALSE NEGATIVES**		
Antifungal prophylaxis and therapy	Low pre-test probability of IFI	Lower median BDG values were reported in breakthrough IFI episodes [16]. BDG should be used to exclude rather than for diagnosis in these patients [18].
Sanctuary sites or poorly vascularized sites of infection	BDG not released into blood	
*Candida parapsilosis* or *Candida auris*	Lower content of BDG component in fungal wall	Lower levels of BDG reported [19,26].
Hyperbilirubinemia	Interference with test procedure	Possible interference, particularly for colorimetric assays [22].

**Table 3:** The performance of BDG assays in recent studies on pneumocystosis in patients with haematological malignancies (HM).

Study	Patients	Comparison	Cut-Off, pg/mL	Sensitivity % (95% CI)	Specificity % (95% CI)	PPV (95% CI)	NPV (95% CI)
Engsbro et al., 2019 [47]	N = 45, HIV, SOT, HSCT, HM, solid cancer	BDG compared to immunofluorescence microscopy					
		All patients	60	89 (52–100)	48 (23–72)		
			80	89 (52–100)	65 (38–86)		
		PCR-positive patients	60	100 (16–100)	71 (29–96)		
			80	100 (16–100)	74 (54–89)		
		BDG compared to clinical categorization					
		All patients	60	89 (65–99)	64 (42–81)		
			80	83 (59–96)	74 (54–89)		
		PCR-positive patients	60	87 (59–98)	100 (16–100)		
			80	80 (52–96)	100 (16–100)		
Morjaria et al., 2019 [42]	N = 53 HM, HSCT, solid cancer	BDG performance vs. PCR in PCR-positive cases					
		Definite/Probable PJP	80	87%	84.6%	94.6	68.8
		Definite/Probable PJP	200	70%	100%	100	52
Szvalb et al., 2020 [56]	N = 101 HM, Solid cancer	BDG performance in PCR-positive cases					
			80	53.5 (43.8–62.9)	78.4 (67.7–86.2)	7.8 (4.4–11.2)	98.0 (97.6–98.5)
			200	41.6 (32.5–51.3)	87.8 (78.5–93.5)	10.4 (4.3–16.5)	97.8 (97.4–98.2)
			400	35.6 (27.0–45.4)	93.2 (85.1–97.1)	15.2 (3.8–26.5)	97.7 (97.3–98.1)
Damiani et al., 2021 [53]	N = 39 Systemic autoimmune or inflammatory disorder, SOT, HM, solid cancer	Proven PJP, defined as a positive microscopic detection of *P. jirovecii* in BAL					
		All patients	80	87 (73–94)	97 (87–99)	97 (85–99)	88 (75–95)
		Only HM population	80	64 (35–85)	100 (74–100)	100 (64–100)	73 (48–89)

BDG, serum (1,3)-beta-D glucan; CI, Confidence Interval; HIV, Human Immunodeficiency Virus; SOT, Solid Organ Transplant; HSCT, Hematopoietic Stem Cell Transplantation, HM, Hematologic Malignancy.

**Table 4:** Strengths and limitations of BDG testing in patients with haematological malignancies (HM).

Strengths	Limitations
Almost panfungal assay [4,5]	Not applicable to Mucorales, Blastomyces, and most cryptococci [4,5]
Rapid turnaround time (approx. 1 h) [7,8,9,10,11]	Batch testing required with most assays [7,8,9,10,11]
Several assays available, including a single sample format [7,8,9,10,11]	Not specific for any fungus and thus needs to be used in combination with other diagnostic methods for identification of species (GM, PCR, radiology, etc.) [1]
Used both for screening and targeted testing [1]	Cut-off provided by manufacturers might need optimizing for better performance [12,13]
In haematological patients, high specificity of two consecutive positive tests [31,32,33]	The need to use glucan-free laboratory materials [15]
More sensitive than blood cultures for deep-seated candidiasis [1]	Possibility of false positive results (see Table 2) [2,15,16,17,18,19,20,21,22,23,24,25,26,27]
High sensitivity for PJP [53,54,55]	Lower sensitivity in patients with haematologic malignancies and IFD compared to other patient groups [16,18]
The only non-invasive test to support the diagnosis of PJP, especially in situations where critical illness precludes invasive diagnostic procedures such as BAL [42]	Of limited use in paediatric population [68,69,70]
Possibility of use in other sterile fluids such as cerebrospinal fluid for fungal central nervous system infections [64,65]	In case of invasive candidiasis, lower sensitivity in case of certain species, such as *C. parapsilosis* or *C. auris* [19,26]
	Not applicable for use in BAL due to high rate of false positive results [66,67]
	Unpredictable rate of decline, unsuitable for rapid evaluation of treatment response [73,74,75,76,77,78]
